# Post-Diagnosis Decline in Moderate-to-Vigorous Physical Activity Is Associated with Higher Triglyceride and Fasting Glucose Levels in Newly Diagnosed Diabetes: A National Cohort Study

**DOI:** 10.3390/jcm15093201

**Published:** 2026-04-22

**Authors:** Byeongsu Kim, Dong Ok Kim, Seogsong Jeong, Hwamin Lee

**Affiliations:** Department of Biomedical Informatics, Korea University College of Medicine, 161 Jeongneung-ro, Seongbuk-gu, Seoul 02708, Republic of Korea; aqudtn@korea.ac.kr (B.K.); dkim1214@korea.ac.kr (D.O.K.); seogsongjeong@gmail.com (S.J.)

**Keywords:** diabetes, physical activity, metabolic health, moderate-to-vigorous physical activity, triglycerides, fasting glucose, cohort study, metabolic indicators

## Abstract

**Background/Objectives:** Evidence remains limited on how post-diagnosis changes in moderate-to-vigorous physical activity (MVPA) are associated with triglyceride and fasting glucose levels in newly diagnosed diabetes. We examined this association in a Korean national cohort. **Methods:** Using the National Health Insurance Service National Sample Cohort, we identified adults with newly diagnosed diabetes in 2009–2010 who completed health screenings in both 2010–2011 (period I) and 2012–2013 (period II). Period II MVPA frequency was examined within strata defined by period I MVPA category. Adjusted least-squares means for five metabolic indicators were estimated using multivariable linear regression. **Results:** Among 3719 participants, the clearest associations were observed among those performing MVPA five or more times per week during period I, in whom lower period II MVPA frequency was associated with higher triglyceride (P for trend = 0.036) and fasting glucose (P for trend = 0.015) levels. Increases in MVPA among initially inactive participants were not consistently associated with favorable metabolic profiles. **Conclusions:** A post-diagnosis decline in MVPA was associated with higher triglyceride and fasting glucose levels, particularly among initially active individuals. Preventing declines in MVPA after diabetes diagnosis may be clinically relevant.

## 1. Introduction

Diabetes mellitus (DM) has emerged as a significant global health concern. According to the Global Burden of Disease Study 2021, approximately 529 million people were living with diabetes worldwide, with the global prevalence projected to exceed 1.3 billion by 2050 [1]. In South Korea, diabetes remains highly prevalent. According to the most recent national fact sheet, the prevalence of diabetes among adults aged ≥30 years was 15.5% in 2021–2022, while prediabetes affected 41.1% of the same population [2].

DM is a chronic metabolic disease characterized by hyperglycemia resulting from defects in insulin secretion, insulin action, or both. Individuals with DM have a substantially higher risk of cardiovascular disease than those without DM and remain at increased risk of microvascular and macrovascular complications, including retinopathy, kidney disease, and lower-extremity complications [3,4]. Despite improvements in awareness and treatment, glycemic control remains suboptimal in Korean adults with diabetes. In the most recent national fact sheet, 32.4% of adults with diabetes achieved an HbA1c level below 6.5%, and only 15.9% met integrated targets for glycemic, blood pressure, and lipid control [2].

In addition to pharmacological treatment, lifestyle modification remains a central component of diabetes management [5]. Alongside dietary modification, regular physical activity remains a key component of diabetes management. Current guidelines recommend at least 150 min of moderate-to-vigorous physical activity (MVPA) weekly, optimally distributed across daily sessions [5,6]. A meta-analysis reported that patients with DM with higher physical activity levels had risk ratios of 0.62 (95% confidence interval [CI], 0.49–0.78) and 0.51 (95% CI, 0.32–0.81) for overall and cardiovascular disease mortality, respectively, compared with physically inactive patients [7]. More recent evidence has further reinforced that structured physical activity improves glycemic control in adults with type 2 diabetes and that exercise dose and modality meaningfully influence cardiometabolic outcomes [8]. A recent dose–response meta-analysis further demonstrated that increased physical activity was associated with reduced cardiovascular morbidity and mortality in individuals with diabetes, with benefits observed across a range of activity levels [9]. In a Korean nationwide cohort, patients with diabetes who engaged in physical activity 5–6 times per week showed the lowest mortality risk, although the estimate was not statistically significant [10]. Recent Korean prospective cohort studies have further suggested that behavioral transitions in physical activity, particularly becoming newly inactive, are associated with higher metabolic syndrome risk [11]. Other studies have addressed physical activity frequency with respect to pneumonia and infection risks and its persistence concerning major adverse cardiovascular events [12,13]. Beyond glycemic control, physical activity has also been associated with favorable changes in lipid metabolism. Habitual physical activity patterns have been linked to lower triglyceride–glucose index values, a composite surrogate marker reflecting both triglyceride and fasting glucose levels [14]. These findings suggest that the metabolic benefits of regular physical activity in diabetes extend beyond glucose regulation to include lipid-related pathways. A recent nationwide cohort study also reported that favorable lifestyle changes, including increased physical activity, were associated with diabetes remission in patients with new-onset type 2 diabetes [15].

Despite the well-established benefits of physical activity in diabetes management, the existing literature has several important gaps. First, most prior studies have examined the association between static physical activity levels and metabolic outcomes, rather than evaluating how changes in physical activity behavior after diagnosis are associated with metabolic indicators [8,11]. Second, although recent studies have examined physical activity changes or trajectories in relation to cardiovascular events [16] or mortality [17] among individuals with type 1 diabetes, evidence linking post-diagnosis changes in physical activity to specific metabolic indicators such as triglyceride and fasting glucose levels remains scarce. Third, while the relationship between physical activity and glycemic control has been extensively studied, comparatively fewer observational studies have specifically examined how post-diagnosis changes in physical activity are associated with triglyceride and fasting glucose levels in adults with newly diagnosed diabetes.

To address these gaps, we examined the association between changes in MVPA frequency and five metabolic health indicators (waist circumference, triglycerides, high-density lipoprotein cholesterol, systolic blood pressure, and fasting glucose) in a Korean national cohort of adults with newly diagnosed diabetes, with particular attention to the metabolic consequences of post-diagnosis declines in MVPA among initially active individuals.

## 2. Materials and Methods

### 2.1. Study Population

The Korean National Health Insurance Service (NHIS) provides universal health coverage for approximately 97% of the Korean population and includes biennial health screening examinations for adults aged 20 years and older [18]. The NHIS database contains sociodemographic information, healthcare utilization records, prescription data, laboratory results, and health behavior data. This study used the NHIS National Sample Cohort (NHIS-NSC), a population-based retrospective cohort comprising approximately 2.2% nationally representative random samples of all NHIS enrollees, with data spanning from 2002 to 2013. The NHIS-NSC includes longitudinal records of health screening results, insurance claims, diagnosis codes, and prescription histories, and has been widely used in epidemiological research on chronic diseases in the Korean population [18,19].

**Figure 1:**
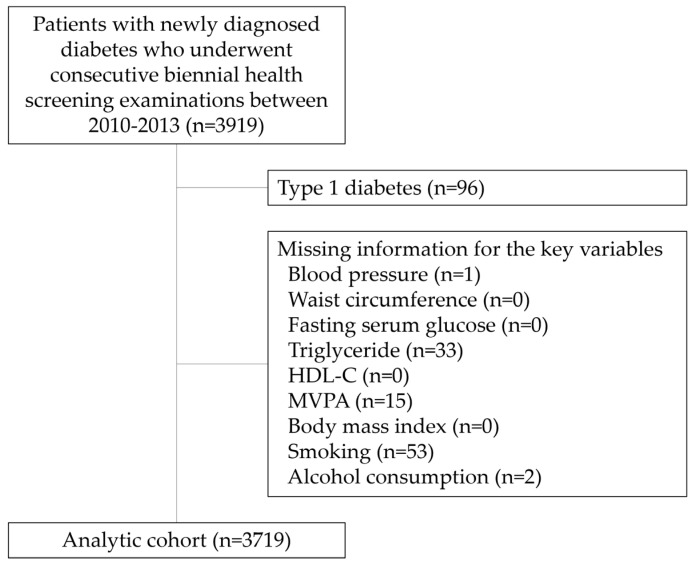
Flowchart of cohort selection. This flowchart illustrates the cohort selection process from the National Health Insurance Service (NHIS) Health Screening Cohort. A total of 3919 individuals newly diagnosed with diabetes mellitus in 2009–2010 were initially identified. Individuals with type 1 diabetes mellitus (*n* = 96) and those with missing key variable information (*n* = 104) were excluded. The final analytic cohort included 3719 participants who underwent health screening examinations during both period I (2010–2011) and period II (2012–2013).

This was a retrospective cohort study using the NHIS-NSC. A total of 3919 individuals with newly diagnosed diabetes in 2009–2010 were initially identified from the NHIS database. Individuals with type 1 diabetes were excluded (*n* = 96). To evaluate post-diagnosis changes in physical activity, we included participants who underwent consecutive biennial health screening examinations in both 2010–2011 (health screening period I) and 2012–2013 (health screening period II). Health screening period I served as the baseline assessment, and health screening period II served as the follow-up assessment for both exposure and outcome measurement. Participants with missing data for key variables were excluded (*n* = 104), resulting in a final analytic sample of 3719 participants (Figure 1). This study was approved by the Institutional Review Board of Korea University Guro Hospital (Approval No. 2024GR0185), and the requirement for informed consent was waived because the data were de-identified.

### 2.2. DM Diagnosis

In this study, newly diagnosed diabetes was operationally defined based on the concurrent presence of International Classification of Diseases, 10th Revision (ICD-10) diagnosis codes E11 (type 2 diabetes mellitus), E12 (malnutrition-related diabetes mellitus), or E14 (unspecified diabetes mellitus) and prescription records for antidiabetic medications during the ascertainment window of 2009–2010. Both conditions (a recorded ICD-10 diagnosis code and at least one prescription for an antidiabetic medication) were required to be met within the same ascertainment window to reduce the possibility of capturing rule-out or incidentally coded diagnoses. Individuals with evidence of diabetes before the 2009–2010 ascertainment window (i.e., any recorded ICD-10 codes E11, E12, or E14 or antidiabetic prescriptions prior to 1 January 2009) were excluded to restrict the cohort to newly diagnosed cases.

### 2.3. Key Variables

The primary exposure was the change in moderate-to-vigorous physical activity (MVPA) frequency between two post-diagnosis health screening periods: period I (2010–2011) and period II (2012–2013). MVPA was assessed using the NHIS health screening questionnaire, which is routinely administered as part of the Korean national health screening program. The questionnaire includes self-reported weekly frequency of moderate physical activity (e.g., fast walking, tennis, and bicycle riding for at least 30 min/day) and vigorous physical activity (e.g., running, aerobics, fast cycling, and mountain hiking for at least 20 min/day). Each item was recorded as the number of days per week, with responses ranging from 0 to 7, and weekly MVPA frequency was calculated as the sum of self-reported moderate and vigorous physical activity days. Weekly MVPA frequency was then categorized into four groups: physically inactive (0 days/week), 1–2 times/week, 3–4 times/week, and ≥5 times/week. The analysis examined period II MVPA frequency within strata defined by period I MVPA category, rather than modeling a single continuous change score.

The study outcomes were five metabolic health indicators measured at health screening period II: waist circumference (cm), triglycerides (mg/dL), high-density lipoprotein cholesterol (mg/dL), systolic blood pressure (mmHg), and fasting glucose (mg/dL). Waist circumference and blood pressure were measured during the health screening examination following standardized protocols. Triglycerides, high-density lipoprotein cholesterol, and fasting glucose were obtained from venous blood samples collected after an overnight fast. The corresponding baseline value of each metabolic indicator measured at period I was included as an adjustment variable in the regression models to account for pre-existing differences in metabolic status. Demographic, behavioral, and clinical covariates measured at period I included age, sex, household income, smoking, alcohol consumption, body mass index (BMI), antihypertensive drug use, lipid-lowering drug use, and Charlson comorbidity index (CCI) [20]. Covariates were assessed at period I rather than period II, as period II values may lie on the causal pathway between changes in MVPA and metabolic outcomes, and adjusting for such variables could introduce overadjustment bias.

### 2.4. Statistical Analysis

Baseline characteristics are presented as numbers (%) for categorical variables and means (standard deviations) for continuous variables. Adjusted mean values and standard errors for metabolic health indicators at period II were estimated using multivariable linear regression models within strata defined by period I MVPA category. In each stratum, period II MVPA category was entered as a categorical independent variable, and adjusted least-squares means were estimated for each category. Pairwise comparisons between period II MVPA categories were conducted with Tukey adjustment for multiple comparisons. P for trend was obtained by entering the period II MVPA category as an ordinal variable in a separate model. The models were adjusted for age, sex, household income, BMI, smoking, alcohol consumption, CCI, and the corresponding baseline value of each metabolic indicator. Subgroup analyses were pre-specified as exploratory and were performed among participants performing MVPA ≥ 5 times/week during period I to explore whether the association between post-diagnosis declines in MVPA and metabolic health indicators differed according to age (<65 vs. ≥65 years), sex, BMI (<25 vs. ≥25 kg/m^2^), smoking, alcohol consumption, antihypertensive drug use, lipid-lowering drug use, and CCI. Potential effect modification was assessed by including a product term between each subgroup variable and the period II MVPA category in the regression model. Given the exploratory nature of the subgroup analyses, these findings should be interpreted as hypothesis-generating. All statistical analyses were conducted using SAS version 9.4 (SAS Institute, Cary, NC, USA). A two-sided *p* value of less than 0.05 was considered statistically significant.

## 3. Results

The cohort selection process is illustrated in Figure 1. The baseline characteristics of the 3719 participants are summarized in Table 1. During period I (2010–2011), 49.5% of participants reported no MVPA, which decreased slightly to 47.4% during period II (2012–2013). The mean age was 59.8 years, 61.7% were male, and 63.4% belonged to the upper half of household income. The mean BMI was 25.2 kg/m^2^, and the mean fasting glucose level was 135.4 mg/dL. Most participants did not consume alcohol (57.0%) or smoke (56.2%). Antihypertensive and lipid-lowering medications were used by 62.7% and 47.0% of participants, respectively. Most participants had a CCI score of 1–2 (44.0%) or 3–4 (39.0%).

The clearest associations were observed among participants performing MVPA ≥ 5 times/week during health screening period I, in whom lower MVPA frequency during period II was associated with higher triglyceride (P for trend = 0.036) and fasting glucose (P for trend = 0.015) levels (Table 2). Specifically, among participants performing MVPA ≥ 5 times/week during period I, those who maintained MVPA ≥ 5 times/week during period II had adjusted mean triglyceride levels of 142.7 mg/dL (SE 5.0), compared with 170.4 mg/dL (SE 9.7) for those who reduced to 1–2 times/week (P = 0.035) and 154.3 mg/dL (SE 5.6) for those who became inactive (P = 0.334). For fasting glucose, those maintaining MVPA ≥ 5 times/week had adjusted mean levels of 122.5 mg/dL (SE 1.9), compared with 128.6 mg/dL (SE 2.2) for those who became inactive (P = 0.099). In contrast, increases in MVPA frequency among initially inactive participants were not consistently associated with more favorable metabolic indicators. Median values and crude mean levels of the metabolic indicators are presented in Supplementary Table S1 and Supplementary Table S2, respectively. In sex-stratified analyses, no consistent differences were observed in male participants (Supplementary Table S3), whereas higher triglyceride levels were observed in female participants with lower follow-up MVPA frequency (Supplementary Table S4).

Subgroup analyses among participants performing MVPA ≥ 5 times/week during health screening period I are presented in Table 3 for triglyceride levels and in Table 4 for fasting glucose levels. For triglyceride levels, a significant interaction was observed only according to antihypertensive drug use (P for interaction = 0.029). For fasting glucose, significant interactions were observed according to alcohol consumption (P for interaction = 0.009) and antihypertensive drug use (P for interaction = 0.014) (Table 4). Because these subgroup analyses were exploratory, the findings should be interpreted cautiously.

## 4. Discussion

This study examined the association between changes in MVPA frequency and metabolic health indicators in adults with newly diagnosed diabetes. The clearest associations were observed among participants performing MVPA ≥ 5 times/week at baseline, in whom lower follow-up MVPA frequency was associated with higher triglyceride and fasting glucose levels. Significant subgroup interactions were observed according to alcohol consumption and antihypertensive drug use; however, these findings should be interpreted cautiously because the subgroup analyses were exploratory. These findings suggest that preventing post-diagnosis declines in MVPA may be clinically relevant, particularly among individuals who are initially physically active. It should be noted that the present study did not have access to information on dietary habits or other lifestyle factors beyond smoking and alcohol consumption, nor did the database include detailed clinical data on comorbidities beyond those captured by the Charlson comorbidity index. These data limitations should be considered when interpreting the observed associations.

Regular physical activity is a central component of diabetes management and has been consistently associated with more favorable glycemic and metabolic profiles in adults with type 2 diabetes [8,21]. Our finding that lower MVPA frequency was associated with higher fasting glucose levels is broadly consistent with prior evidence suggesting that habitual activity level and exercise dose are relevant to glucose control, as well as with recent Korean cohort data linking unfavorable physical activity transitions to higher metabolic syndrome risk [11]. Several mechanisms have been proposed to partially explain the association between physical activity and glycemic control. These include enhanced glucose transporter type 4 (GLUT4) translocation in skeletal muscle, improved peripheral insulin sensitivity, and increased mitochondrial oxidative capacity, although the magnitude and clinical relevance of these effects may vary depending on exercise type, intensity, and individual metabolic status [22,23]. Accordingly, these pathways may contribute to, but do not fully account for, the observed association. However, direct evidence specifically examining reductions in MVPA after diabetes diagnosis remains limited.

Our triglyceride findings are also broadly consistent with previous evidence suggesting that aerobic and moderate-intensity physical activity may improve lipid utilization and reduce fasting triglyceride levels compared with inactivity [24,25]. In the present study, among participants performing MVPA ≥ 5 times/week during period I, those who maintained the same level during period II had adjusted mean triglyceride levels of 142.7 mg/dL, compared with 170.4 mg/dL for those who reduced to 1–2 times/week (P = 0.035, Table 2). More recent evidence linking physical activity patterns to the triglyceride–glucose index further supports the relevance of habitual activity patterns to adverse glucose–lipid metabolic burden [14]. However, direct evidence specifically addressing post-diagnosis reductions in MVPA among adults with type 2 diabetes remains limited.

The interaction between alcohol consumption and fasting glucose levels should be interpreted cautiously. Although previous evidence has suggested that alcohol intake may be associated with impaired fasting glycemia risk [26], a recent large-scale prospective cohort study reported that the association between alcohol consumption and type 2 diabetes risk varied substantially by drinking frequency and pattern, underscoring the importance of detailed assessment of alcohol intake in metabolic research [27]. Our study did not include quantitative measures of alcohol intake and could only distinguish between drinkers and non-drinkers, precluding a more detailed evaluation of frequency- or dose-dependent effects. This finding should therefore be regarded as hypothesis-generating rather than definitive.

The subgroup findings should be interpreted cautiously because they were exploratory. Our results may be better interpreted as reflecting the clinical relevance of behavioral transitions in physical activity rather than the effect of a single static activity category.

This study has several limitations. MVPA was assessed by self-report using the NHIS health screening questionnaire, which captures only the weekly frequency of moderate and vigorous physical activity without recording duration or intensity per session. This approach does not align directly with guideline-based recommendations expressed in minutes per week (e.g., ≥150 min/week of MVPA) and may have introduced exposure misclassification [5,6]. However, the same frequency-based measure has been widely used in prior epidemiological studies with this cohort [10,12,13]. The questionnaire did not distinguish aerobic from resistance exercise, and sedentary behavior was not assessed [28,29]. In addition, the database did not provide detailed information on medication type, dosage, dietary habits, or alcohol quantity; alcohol consumption could only be classified as a binary variable (drinkers vs. non-drinkers), and information on education level was not available, although household income was included as a proxy for socioeconomic status.

The temporal structure of the study also warrants consideration. Because the primary exposure (change in MVPA between period I and period II) and the outcomes were both assessed at period II, the possibility of reverse causation cannot be fully excluded, and the findings should be interpreted as associations rather than causal effects. Furthermore, individuals who reduced their MVPA after diagnosis may have differed systematically from those who maintained activity levels in ways not fully captured by the available covariates, and residual confounding cannot be excluded despite adjustment for age, sex, income, BMI, smoking, alcohol consumption, and CCI.

This study used the NHIS-NSC, which is a closed cohort with data available from 2002 to 2013. The analytic time window was therefore constrained by the structure of the cohort database rather than by investigator choice. As diabetes management practices and physical activity monitoring have evolved since the study period, the generalizability of these findings to contemporary clinical settings should be considered with caution. In addition, the principal contrasts in this study were centered on clinically distinct baseline MVPA categories, and the findings should be interpreted as reflecting selected behavioral transitions rather than a uniform dose–response pattern across all activity levels [11]. Despite these limitations, this study provides national cohort evidence that post-diagnosis declines in MVPA, particularly among initially active individuals, were associated with higher triglyceride and fasting glucose levels.

Although increases in MVPA were not consistently associated with more favorable metabolic profiles, baseline physical inactivity may have limited the extent of observable benefit. In contrast, lower MVPA frequency after diagnosis was associated with higher triglyceride and fasting glucose levels, with the clearest pattern observed among participants who were initially physically active. These findings are aligned with current diabetes care recommendations emphasizing regular physical activity as a core component of long-term disease management. Future studies incorporating device-measured physical activity data, sedentary behavior, time-varying covariate adjustment, and more detailed lifestyle measures are needed to refine exercise recommendations after diabetes diagnosis.

## 5. Conclusions

In this national cohort study of adults with newly diagnosed diabetes, a decline in MVPA frequency after diagnosis was associated with higher triglyceride and fasting glucose levels, with the clearest pattern observed among participants who were initially physically active. In contrast, increases in MVPA among initially inactive participants were not consistently associated with more favorable metabolic profiles. These findings highlight the potential clinical importance of preventing post-diagnosis declines in physical activity, particularly among individuals who are already engaging in regular MVPA at the time of diagnosis. The study was limited by the use of self-reported, frequency-based MVPA data and the reliance on administrative health screening records from 2010 to 2013. Future studies using device-measured physical activity, incorporating time-varying lifestyle and pharmacological covariates, and employing prospective longitudinal designs are warranted to further clarify the metabolic consequences of behavioral transitions in physical activity following a diabetes diagnosis.

## Figures and Tables

**Table 1:** Baseline Characteristics of Participants in the National Health Insurance Service (NHIS) Health Screening Cohort.

Characteristic	Participants (*n* = 3719)
MVPA during health screening period I (2010–2011)	
0	1840 (49.5)
1–2 times/week	563 (15.1)
3–4 times/week	518 (13.9)
≥5 times/week	798 (21.5)
MVPA during health screening period II (2012–2013)	
0	1762 (47.4)
1–2 times/week	517 (13.9)
3–4 times/week	495 (13.3)
≥5 times/week	945 (25.4)
Age, years	59.8 (8.5)
Sex, *n* (%)	
Male	2296 (61.7)
Female	1423 (38.3)
Household income ^a^, *n* (%)	
Upper half	2358 (63.4)
Lower half	1361 (36.6)
Body mass index, kg/m^2^	25.2 (3.1)
Waist circumference, cm	85.8 (7.9)
Systolic blood pressure, mmHg	128.7 (14.8)
Diastolic blood pressure, mmHg	79.4 (9.6)
Total cholesterol, mg/dL	202.4 (42.7)
High-density lipoprotein cholesterol, mg/dL	51.6 (20.8)
Triglycerides, mg/dL	178.8 (121.5)
Fasting glucose, mg/dL	135.4 (46.6)
Alcohol consumption, *n* (%)	
No	2118 (57.0)
Yes	1601 (43.0)
Cigarette smoking, *n* (%)	
No	2089 (56.2)
Yes	1630 (43.8)
Antihypertensive drugs, *n* (%)	2332 (62.7)
Lipid-lowering drugs, *n* (%)	1748 (47.0)
Charlson comorbidity index, *n* (%)	
1–2	1636 (44.0)
3–4	1451 (39.0)
≥5	632 (17.0)

Continuous data are presented as means (standard deviations). Categorical data are expressed as numbers (%). ^a^ Proxy for socioeconomic status based on the insurance premium of the National Health Insurance Service. Abbreviations: MVPA, moderate-to-vigorous physical activity; HDL-C, high-density lipoprotein cholesterol; CCI, Charlson comorbidity index.

**Table 2:** Adjusted Mean Values of Period II Metabolic Indicators According to Changes in Weekly MVPA Frequency Between Period I and Period II.

	WC, cm		TG, mg/dL		HDL-C, mg/dL		SBP, mmHg		FG, mg/dL	
	Mean (SE)	*P*	Mean (SE)	*P*	Mean (SE)	*P*	Mean (SE)	*P*	Mean (SE)	*P*
Period I: No MVPA										
Period II MVPA										
None	85.2 (0.2)		155.3 (2.9)		51.1 (0.4)		127.4 (0.5)		122.0 (1.0)	
1–2	85.4 (0.3)	0.922	152.4 (5.9)	0.969	50.6 (0.8)	0.929	126.4 (0.9)	0.773	118.6 (2.1)	0.426
3–4	84.8 (0.4)	0.820	145.8 (7.3)	0.605	52.6 (1.0)	0.483	128.7 (1.2)	0.685	119.0 (2.6)	0.682
≥5	84.9 (0.3)	0.746	147.2 (5.1)	0.464	51.3 (0.7)	0.997	126.9 (0.8)	0.964	120.4 (1.8)	0.836
*P* trend		0.491		0.370		0.433		0.432		0.350
Period I: MVPA ≥5 times/week										
Period II MVPA										
≥5	84.6 (0.3)		142.7 (5.0)		52.3 (0.7)		126.1 (0.8)		122.5 (1.9)	
3–4	84.7 (0.5)	1.000	146.0 (7.5)	0.978	51.8 (1.1)	0.978	127.5 (1.2)	0.768	118.0 (2.9)	0.496
1–2	86.1 (0.6)	0.107	170.4 (9.7)	0.035	52.8 (1.4)	0.984	126.6 (1.6)	0.992	121.6 (3.7)	0.996
None	85.0 (0.4)	0.888	154.3 (5.6)	0.334	51.2 (0.8)	0.720	127.4 (0.9)	0.691	128.6 (2.2)	0.099
*P* trend		0.147		0.036		0.673		0.649		0.015

Means and *p* values are calculated using multivariable linear regression after adjustments for age, sex, household income, body mass index, smoking, alcohol consumption, Charlson comorbidity index, and the corresponding baseline metabolic indicator. Pairwise comparisons were conducted with Tukey adjustment. P trend was obtained by entering the period II MVPA category as an ordinal variable. Period II MVPA frequency is expressed as times/week. Abbreviations: WC, waist circumference; TG, triglycerides; HDL-C, high-density lipoprotein cholesterol; SBP, systolic blood pressure; FG, fasting glucose; MVPA, moderate-to-vigorous physical activity; CCI, Charlson comorbidity index.

**Table 3:** Subgroup Analyses of Adjusted Triglyceride Levels According to MVPA Frequency During Period II (2012–2013) Among Participants performing MVPA ≥ 5 Times/Week During Period I (2010–2011).

Subgroup	Period II MVPA					
	≥5	3–4	1–2	None	*P* Trend	*P* *int.*
Age, years						0.663
<65	144.3 (6.2)	151.2 (9.0)	172.3 (11.9)	153.7 (7.6)	0.147	
≥65	136.4 (8.4)	124.6 (13.4)	165.3 (15.5)	147.4 (7.5)	0.159	
BMI, kg/m^2^						0.065
<25	129.4 (7.4)	146.1 (11.5)	181.4 (14.6)	150.3 (8.9)	0.005	
≥25	157.7 (6.8)	145.6 (9.8)	161.3 (13.0)	158.7 (7.2)	0.654	
Smoking						0.821
No	127.3 (5.7)	133.0 (8.7)	161.1 (11.1)	142.9 (5.8)	0.023	
Yes	134.1 (23.8)	135.1 (26.0)	157.1 (27.9)	140.4 (24.3)	0.571	
Alcohol						0.950
No	133.9 (6.1)	139.8 (10.4)	166.8 (11.8)	145.6 (6.7)	0.066	
Yes	146.8 (10.7)	146.3 (13.2)	169.3 (16.7)	156.7 (11.4)	0.486	
Antihypertensive						0.029
No	140.8 (10.3)	163.5 (14.8)	203.4 (20.3)	159.7 (12.8)	0.020	
Yes	145.1 (5.3)	136.4 (8.0)	151.7 (10.1)	151.6 (5.7)	0.415	
Lipid-lowering						0.088
No	140.3 (8.0)	150.3 (12.1)	197.1 (16.0)	154.2 (9.2)	0.008	
Yes	145.4 (6.2)	143.0 (9.0)	150.2 (11.5)	155.3 (6.9)	0.635	
CCI						0.338
1–2	138.1 (7.9)	154.6 (11.9)	186.0 (16.8)	147.8 (9.7)	0.045	
3–4	151.2 (7.2)	135.6 (11.7)	160.4 (14.0)	155.8 (8.5)	0.450	
≥5	132.4 (11.6)	139.7 (16.2)	155.8 (21.1)	160.0 (10.7)	0.256	

Values are presented as means (standard errors). Means and *p* values are calculated using multivariable linear regression after adjustments for age, sex, household income, body mass index, smoking, alcohol consumption, Charlson comorbidity index, and the corresponding baseline metabolic indicator. P for interaction was assessed by including a product term between each subgroup variable and the period II MVPA category. Period II MVPA frequency is expressed as times/week. P trend, P for trend; P int., P for interaction. Abbreviations: BMI, body mass index; CCI, Charlson comorbidity index; MVPA, moderate-to-vigorous physical activity.

**Table 4:** Subgroup Analyses of Adjusted Fasting Glucose Levels According to MVPA Frequency During Period II (2012–2013) Among Participants performing MVPA ≥5 Times/Week During Period I (2010–2011).

Subgroup	Period II MVPA					
	≥5	3–4	1–2	None	*P* Trend	*P* *int.*
Age, years						0.728
<65	124.4 (2.4)	119.1 (3.5)	123.7 (4.6)	131.8 (2.9)	0.024	
≥65	115.6 (3.1)	114.5 (5.1)	115.5 (5.9)	117.8 (2.9)	0.912	
BMI, kg/m^2^						0.348
<25	122.3 (2.5)	114.3 (3.9)	125.9 (5.0)	127.3 (3.1)	0.048	
≥25	122.2 (3.0)	121.7 (4.2)	117.3 (5.6)	129.3 (3.1)	0.153	
Smoking						0.849
No	120.2 (2.6)	114.4 (4.0)	121.9 (5.0)	125.4 (2.6)	0.125	
Yes	126.2 (8.2)	122.8 (8.9)	122.3 (9.6)	133.2 (8.4)	0.153	
Alcohol						0.009
No	123.1 (2.4)	116.2 (4.2)	112.7 (4.8)	123.8 (2.7)	0.086	
Yes	123.9 (3.9)	121.8 (4.9)	134.3 (6.2)	137.2 (4.2)	0.003	
Antihypertensive					0.014	
No	124.1 (3.8)	119.5 (5.4)	129.3 (7.5)	142.1 (4.7)	0.002	
Yes	122.7 (2.1)	118.1 (3.2)	117.9 (4.1)	122.3 (2.3)	0.459	
Lipid-lowering						0.168
No	124.0 (3.0)	118.0 (4.5)	118.2 (6.0)	134.4 (3.4)	0.005	
Yes	121.3 (2.5)	119.0 (3.6)	124.7 (4.6)	122.4 (2.7)	0.758	
CCI					0.858	
1–2	126.2 (2.9)	122.1 (4.4)	130.7 (6.2)	131.9 (3.5)	0.293	
3–4	125.3 (3.1)	121.5 (5.1)	119.1 (6.1)	133.2 (3.7)	0.105	
≥5	115.9 (3.4)	108.8 (4.8)	118.6 (6.2)	119.3 (3.2)	0.285	

Values are presented as means (standard errors). Means and *p* values are calculated using multivariable linear regression after adjustments for age, sex, household income, body mass index, smoking, alcohol consumption, Charlson comorbidity index, and the corresponding baseline metabolic indicator. P for interaction was assessed by including a product term between each subgroup variable and the period II MVPA category. Period II MVPA frequency is expressed as times/week. P trend, P for trend; P int., P for interaction. Abbreviations: BMI, body mass index; CCI, Charlson comorbidity index; MVPA, moderate-to-vigorous physical activity.

## Data Availability

The data used in this study are available from the National Health Insurance Service (NHIS) Data Sharing Service under license and are not publicly available. Data access is subject to NHIS approval.

## Supplementary Materials

The following supporting information can be downloaded at: https://www.mdpi.com/article/10.3390/jcm15093201/s1, Table S1: Median Values of Period II Metabolic Indicators According to Changes in Weekly MVPA Frequency Between Period I and Period II; Table S2: Crude Mean Values of Period II Metabolic Indicators According to Changes in Weekly MVPA Frequency Between Period I and Period II; Table S3: Adjusted Mean Values of Period II Metabolic Indicators According to Changes in Weekly MVPA Frequency Between Period I and Period II Among Male Participants; Table S4: Adjusted Mean Values of Period II Metabolic Indicators According to Changes in Weekly MVPA Frequency Between Period I and Period II Among Female Participants.
