# Supplementary material for: Post-Diagnosis Decline in Moderate-to-Vigorous Physical Activity Is Associated with Higher Triglyceride and Fasting Glucose Levels in Newly Diagnosed Diabetes: A National Cohort Study
Source: J Clin Med. 2026 Apr 22;15(9):3201. doi: 10.3390/jcm15093201 (PMC13164427; doi:10.3390/jcm15093201)
Supplement: Supplementary file 1 [file jcm-15-03201-s001.zip › Supplementary Table S1.pdf]

Supplementary Table S1. Median Values of Period II Metabolic Indicators According to Changes in Weekly MVPA Frequency Between Period I and Period II

	Waist circumference, cm	Triglycerides, mg/dL	HDL-C, mg/dL	Systolic blood pressure, mmHg	Fasting serum glucose, mg/dL
No MVPA during health screening period I (2010–2011)					
MVPA during health screening period II (2012–2013)					
None	85 (80–91)	128 (94–187)	49 (42–58)	129 (119–138)	117 (100–133)
1–2 times/week	86 (80–91)	128 (93–189)	50 (43–57)	126 (115–135)	115 (103–133)
3–4 times/week	84 (77–91)	132 (94–178)	50 (43–61)	130 (118–137)	116 (99–133)
≥5 times/week	84 (79–90)	131 (84–177)	49 (41–58)	128 (119–136)	116 (100–133)
MVPA ≥ 5 times/week during health screening period I (2010–2011)					
MVPA during health screening period II (2012–2013)					
≥5 times/week	86 (81–90)	120 (84–162)	50 (42–60)	126 (118–134)	118 (105–136)
3–4 times/week	85 (80–90)	111 (82–177)	50 (43–58)	127 (120–135)	114 (104–132)
1–2 times/week	87 (81–92)	138 (94–214)	50 (42–62)	130 (118–137)	120 (103–130)
None	85 (80–91)	132 (98–187)	48 (42–58)	129 (119–137)	121 (106–142)

Data are expressed as medians (interquartile ranges).

Abbreviations: MVPA, moderate-to-vigorous physical activity; HDL-C, high-density lipoprotein cholesterol
