# Supplementary material for: Post-Diagnosis Decline in Moderate-to-Vigorous Physical Activity Is Associated with Higher Triglyceride and Fasting Glucose Levels in Newly Diagnosed Diabetes: A National Cohort Study
Source: J Clin Med. 2026 Apr 22;15(9):3201. doi: 10.3390/jcm15093201 (PMC13164427; doi:10.3390/jcm15093201)
Supplement: Supplementary file 1 [file jcm-15-03201-s001.zip › Supplementary Table S2.pdf]

Supplementary Table S2. Crude Mean Values of Period II Metabolic Indicators According to Changes in Weekly MVPA Frequency Between Period I and Period II

	Waist circumference, cm		Triglycerides, mg/dL		HDL-C, mg/dL		Systolic blood pressure, mmHg		Fasting serum glucose, mg/dL	
	**Mean (SE)**	***P* value**	**Mean (SE)**	***P* value**	**Mean (SE)**	***P* value**	**Mean (SE)**	***P* value**	**Mean (SE)**	***P* value**
No MVPA during health screening period I (2010–2011)										
MVPA during health screening period II (2012–2013)										
None	85.3 (0.2)		154.5 (2.9)		50.6 (0.4)		128.0 (0.4)		121.2 (0.9)	
1–2 times/week	85.6 (0.6)	0.972	156.2 (6.5)	0.995	50.4 (0.8)	0.997	125.8 (1.0)	0.180	120.9 (2.1)	0.999
3–4 times/week	84.5 (0.7)	0.766	153.7 (8.2)	1.000	52.4 (1.1)	0.368	128.2 (1.2)	0.998	121.9 (2.7)	0.996
≥5 times/week	84.6 (0.5)	0.638	145.4 (5.6)	0.471	50.5 (0.7)	1.000	126.8 (0.8)	0.593	121.3 (1.8)	1.000
P for trend		0.455		0.502		0.410		0.158		0.994
MVPA ≥ 5 times/week during health screening period I (2010–2011)										
MVPA during health screening period II (2012–2013)										
≥5 times/week	85.0 (0.4)		137.6 (4.6)		51.4 (0.7)		126.3 (0.7)		123.6 (1.7)	
3–4 times/week	84.8 (0.7)	0.993	139.5 (7.8)	0.997	51.3 (1.1)	1.000	127.6 (1.2)	0.802	119.2 (2.9)	0.558
1–2 times/week	86.5 (1.0)	0.470	167.5 (10.3)	0.041	51.4 (1.5)	1.000	126.5 (1.6)	0.999	121.7 (3.8)	0.972
None	85.7 (0.5)	0.757	151.3 (5.7)	0.246	49.9 (0.8)	0.491	127.8 (0.9)	0.525	128.2 (2.1)	0.326
P for trend		0.373		0.029		0.525		0.538		0.071

Crude means and *P* values are calculated using linear regression.

Abbreviations: MVPA, moderate-to-vigorous physical activity; SE, standard error; HDL-C, high-density lipoprotein cholesterol
