# Supplementary material for: Post-Diagnosis Decline in Moderate-to-Vigorous Physical Activity Is Associated with Higher Triglyceride and Fasting Glucose Levels in Newly Diagnosed Diabetes: A National Cohort Study
Source: J Clin Med. 2026 Apr 22;15(9):3201. doi: 10.3390/jcm15093201 (PMC13164427; doi:10.3390/jcm15093201)
Supplement: Supplementary file 1 [file jcm-15-03201-s001.zip › Supplementary Table S3.pdf]

Supplementary Table S3. Adjusted Mean Values of Period II Metabolic Indicators According to Changes in Weekly MVPA Frequency Between Period I and Period II Among Male Participants

	Waist circumference, cm		Triglycerides, mg/dL		HDL-C, mg/dL		Systolic blood pressure, mmHg		Fasting serum glucose, mg/dL	
	**aMean (SE)**	***P* value**	**aMean (SE)**	***P* value**	**aMean (SE)**	***P* value**	**aMean (SE)**	***P* value**	**aMean (SE)**	***P* value**
No MVPA during health screening period I (2010–2011)										
MVPA during health screening period II (2012–2013)										
None	87.2 (0.2)		161.5 (4.2)		48.5 (0.5)		126.9 (0.6)		125.0 (1.5)	
1–2 times/week	87.4 (0.4)	0.979	149.8 (8.3)	0.551	49.0 (0.9)	0.951	125.9 (1.2)	0.871	122.3 (2.9)	0.824
3–4 times/week	87.4 (0.6)	0.988	158.9 (10.7)	0.995	49.4 (1.2)	0.896	129.7 (1.6)	0.294	118.5 (3.7)	0.327
≥5 times/week	87.3 (0.4)	1.000	150.3 (6.9)	0.458	48.9 (0.8)	0.960	126.6 (1.0)	0.991	122.4 (2.4)	0.767
P for trend		0.973		0.360		0.859		0.220		0.316
MVPA ≥ 5 times/week during health screening period I (2010–2011)										
MVPA during health screening period II (2012–2013)										
≥5 times/week	86.5 (0.4)		142.4 (6.1)		50.1 (0.9)		126.2 (0.9)		121.7 (2.3)	
3–4 times/week	86.8 (0.5)	0.940	149.0 (9.2)	0.919	48.6 (1.3)	0.740	128.2 (1.4)	0.547	118.4 (3.5)	0.823
1–2 times/week	87.9 (0.7)	0.271	164.4 (12.7)	0.353	49.1 (1.8)	0.952	125.3 (2.0)	0.974	120.2 (4.9)	0.991
None	86.7 (0.4)	0.980	149.8 (7.7)	0.853	48.1 (1.1)	0.413	128.0 (1.2)	0.566	128.7 (2.9)	0.195
P for trend		0.345		0.395		0.434		0.336		0.092

aMeans and *P* values are calculated using linear regression after adjustments for age, sex, household income, body mass index, smoking, alcohol consumption, Charlson comorbidity index, and the corresponding baseline metabolic characteristics.

Abbreviations: MVPA, moderate-to-vigorous physical activity; aMean, adjusted mean; SE, standard error; HDL-C, high-density lipoprotein cholesterol
