# Supplementary material for: Post-Diagnosis Decline in Moderate-to-Vigorous Physical Activity Is Associated with Higher Triglyceride and Fasting Glucose Levels in Newly Diagnosed Diabetes: A National Cohort Study
Source: J Clin Med. 2026 Apr 22;15(9):3201. doi: 10.3390/jcm15093201 (PMC13164427; doi:10.3390/jcm15093201)
Supplement: Supplementary file 1 [file jcm-15-03201-s001.zip › Supplementary Table S4.pdf]

**Supplementary Table S4. Adjusted Mean Values of Period II Metabolic Indicators According to Changes in Weekly MVPA Frequency Between Period I and Period II Among Female Participants**

| | Waist circumference, cm | | Triglycerides, mg/dL | | HDL-C, mg/dL | | Systolic blood pressure, mmHg | | Fasting serum glucose, mg/dL | |
|---|---|---|---|---|---|---|---|---|---|---|
| | **aMean (SE)** | ***P* value** | **aMean (SE)** | ***P* value** | **aMean (SE)** | ***P* value** | **aMean (SE)** | ***P* value** | **aMean (SE)** | ***P* value** |
| No MVPA during health screening period I (2010–2011) | | | | | | | | | | |
| MVPA during health screening period II (2012–2013) | | | | | | | | | | |
| None | 82.8 (0.6) | | 148.5 (8.5) | | 56.1 (1.3) | | 126.1 (1.6) | | 124.7 (3.2) | |
| 1–2 times/week | 83.0 (0.8) | 0.976 | 157.3 (11.4) | 0.741 | 54.6 (1.7) | 0.695 | 125.4 (2.1) | 0.962 | 120.4 (4.3) | 0.548 |
| 3–4 times/week | 81.6 (0.9) | 0.349 | 131.6 (12.7) | 0.351 | 58.2 (1.9) | 0.507 | 125.7 (2.3) | 0.995 | 126.2 (4.7) | 0.979 |
| ≥5 times/week | 81.9 (0.7) | 0.352 | 147.3 (10.6) | 0.999 | 55.8 (1.6) | 0.998 | 125.9 (1.9) | 0.998 | 124.1 (4.0) | 0.997 |
| *P* for trend | | 0.138 | | 0.233 | | 0.305 | | 0.965 | | 0.554 |
| MVPA ≥ 5 times/week during health screening period I (2010–2011) | | | | | | | | | | |
| MVPA during health screening period II (2012–2013) | | | | | | | | | | |
| ≥5 times/week | 81.6 (1.5) | | 113.2 (16.1) | | 51.4 (2.6) | | 122.3 (3.5) | | 123.5 (6.7) | |
| 3–4 times/week | 80.3 (1.8) | 0.702 | 107.6 (19.4) | 0.975 | 54.2 (3.1) | 0.886 | 122.0 (4.2) | 1.000 | 114.0 (8.0) | 0.308 |
| 1–2 times/week | 83.7 (1.9) | 0.433 | 147.7 (19.7) | 0.073 | 55.5 (3.2) | 0.997 | 125.9 (4.3) | 0.642 | 122.7 (8.2) | 0.999 |
| None | 82.2 (1.5) | 0.913 | 128.8 (16.0) | 0.336 | 53.8 (2.6) | 0.396 | 122.4 (3.5) | 1.000 | 127.9 (6.6) | 0.666 |
| *P* for trend | | 0.189 | | 0.041 | | 0.201 | | 0.663 | | 0.095 |

aMeans and *P* values are calculated using linear regression after adjustments for age, sex, household income, body mass index, smoking, alcohol consumption, Charlson comorbidity index, and the corresponding baseline metabolic characteristics.

Abbreviations: MVPA, moderate-to-vigorous physical activity; aMean, adjusted mean; SE, standard error; HDL-C, high-density lipoprotein cholesterol
